# Epworth sleepiness scale in medical residents: quality of sleep and its relationship to quality of life

**DOI:** 10.1186/s12995-018-0203-z

**Published:** 2018-07-13

**Authors:** Yehia Z. Alami, Beesan T. Ghanim, Sa’ed H. Zyoud

**Affiliations:** 1 Department of Medicine, College of Medicine and Health Sciences, An-Najah National University, Nablus, 44839 Palestine; 2 Poison Control and Drug Information Center (PCDIC), College of Medicine and Health Sciences, An-Najah National University, Nablus, 44839 Palestine; 3 Department of Clinical and Community Pharmacy, Department of Pharmacy, College of Medicine and Health Sciences, An-Najah National University, Nablus, 44839 Palestine

**Keywords:** Residents, Epworth, Sleepiness, Quality of life, SF-36

## Abstract

**Background:**

Resident doctors are continuously exposed to prolonged working hours and night shifts, making them susceptible to the many physical, psychological, and cognitive side effects of sleep deprivation, which may affect their quality of life. Therefore, this study aimed to determine the prevalence of sleep penury in resident doctors and to assess the association between self-apprehended sleepiness and quality of life.

**Methods:**

A cross-sectional study was carried out in the governmental hospitals in the North of the West Bank between May 2017 and September 2017. Doctors enrolled in residency programmes completed questionnaires about general, sociodemographic, and sleep characteristics. The doctors completed the Arabic Version of the Epworth Sleepiness Scale (ArESS) to assess subjective daytime sleepiness and the RAND 36-item short-form health survey (SF-36) to determine quality of life.

**Results:**

A total of 101 participants were enrolled. Daytime sleepiness was observed in 37.6% (*n* = 38) of the participants with an ESS score of ≥10. There was a notable negative correlation between the ESS and quality of health index in the physical composition (*r* = − 0.351, *p* < 0.001) demonstrated in the following four subscales: the physical functioning (*p* < 0.001), role limitations due to physical health (*p* = 0.045), body pain (*p* = 0.036), and general health (*p* < 0.001) components of the SF-36 scale. Females and residents of the centre region had poorer mental quality (*p* = 0.006 and 0.020, respectively).

**Conclusions:**

More than one third of the resident doctors suffer from daytime sleepiness according to the ESS. This was proven to significantly affect several aspects of their quality of life, including physical function and health, body pain, and general health. Sleep deprivation and improvement of quality of life require health promotion actions among medical residents.

**Electronic supplementary material:**

The online version of this article (10.1186/s12995-018-0203-z) contains supplementary material, which is available to authorized users.

## Background

According to many articles, sleep deprivation affects many physiological and psychological aspects of one’s life [1–3]. Sleep deprivation affects episodic memory as well as self-awareness and responsibility [3–5]. Sleep loss also severely affects the emotional memory in its contextual and non-contextual aspects [6]. Neuronal apoptosis using adrenergic receptors can be triggered due to the deprivation of rapid eye movement (REM) sleep component [7], as well as induce anxiolytic-like effects by dopaminergic influences [8]. According to De Bernardi Rodrigues et al., sleep deprivation is associated with decreased sensitivity to insulin and centripetal distribution of fat in adolescence [9].

One of the many professions that is continuously exposed to prolonged periods of sleep deprivation is that of a resident doctor. The prolonged working hours of resident physicians make them vulnerable to the consequences of sleep penury, which affects their task performance and quality of life [10]. The residents’ poor sleep quality is associated with increased body mass index (BMI), poorer lipid profile, poor quality of food consumed, and increase in waist circumference [11]. The nature of this poor sleep may also affect the residents psychologically by making them depressed, anxious, and insomnious [12–14]. Immune function has also been studied in relation to poor sleep quality, indicating the probability of inflammatory responses, triggered by short sleep duration, producing metabolic, respiratory, and cardiovascular diseases. In addition, it has been reported that poor sleep might cause elevated C-reactive protein levels, an inflammatory marker for cardiovascular diseases [10, 15].

Several studies have demonstrated how sleep deprivation negatively impacts one’s well-being in general and how it negatively influences resident doctors specifically [3, 5, 10, 12–14, 16, 17]. However, to our knowledge, little is known about the effects of daytime sleepiness on residents’ health [16]. Although limited studies have been conducted and published about sleep habits and problems among Palestinian university students [18, 19], Palestinian patients diagnosed with cancer [20], and the Palestinian population [21], no studies have been conducted to focus on the relationship between self-reported sleepiness, as surrogate marker of poor sleep, and quality of life in resident doctors in Palestine. Therefore, the purposes of this investigation are to determine the prevalence of self-reported sleepiness in resident doctors and to assess the association between sleepiness and their quality of life. The significance of this study may be reflected in the rules and policies of the Ministry of Health by proposing new working hours, changing on-call guidelines and duties, strengthening the on-call team, improving sleeping places to promote better sleep hygiene, offering free times for taking naps [16, 22], providing stress-relieving activities, and adopting rewarding programmes i.e. providing extra money and increasing residents’ days-off to galvanise the resident physicians.

## Methods

### Study design and population

A cross-sectional study design was employed to achieve the research objectives. The stud**y** took place amongst a group of health care practitioners, as it included resident doctors who are training in different specialities. Resident doctors who are general practitioners were excluded because they are not in the medical training (residency) programme.

### Setting

The data were collected from governmental hospitals in three cities in the North of the West Bank (Jenin, Nablus, and Tulkarm). These hospitals are the sites of the medical training programme for the resident doctors in North of the West Bank.

### Sample size and sampling technique

The estimated number of resident doctors licensed by the Palestinian Medical Association who worked at the surveyed hospitals was around 130. Accordingly, the Raosoft sample size calculator was used to perceive the sample size [23] by keeping an indicator percentage of 0.50, a margin of error of 5%, and a confidence interval of 95%. The calculated sample size was 98. Between May 2017 and September 2017, we enrolled a convenience sample of 126 resident doctors.

### Data collection

The doctors completed surveys about their sociodemographic, general, and sleep characteristics. Sociodemographic and general characteristics included age, sex, marital status, governorates they come from in the West Bank (north, central, south), who they live with (with family or not), BMI and change in weight during residency, presence of chronic diseases, place of graduation, working department, number of residency years, and years of work experience. Sleep characteristics included questions about sleeping hours on days with and without shifts, caffeine intake, smoking, use of sleeping pills, time to fall asleep, and waking up during sleep.

### Instruments

Physicians were asked to complete two instruments: the Arabic Version of the Epworth sleepiness scale (ArESS) to assess the quality of sleep and the RAND 36-item short-form health survey (SF-36).

### Arabic Version of the Epworth sleepiness scale (ArESS)

The Epworth Sleepiness Scale (ESS) was developed to assess sleepiness during daytime. The ESS questionnaire consists of eight questions about daily situations that can induce sleepiness. Each question has the lowest score of 0 (‘no chance of falling asleep’) to 3 (‘high chance of falling asleep’). The score ranges can sum up to 24 and are categorised as normal (ESS < 10) or positive for daytime sleepiness (ESS ≥ 10) [24]. We used the ArESS, which has been validated and is reliable to be used in populations that are Arabic-speaking [25]. Permission to use the ArESS was given by Professor Hamdan Al-Jahdali via e-mail correspondence. There is a high level of internal consistency within the ArESS in our study, as measured by Cronbach’s alpha (0.749).

### RAND 36-item short-form health survey (SF-36)

This survey was employed to discern the general quality of life based on the previous one month’s experiences [26]. It consists of 36 items divided into subscales, each with its own score: physical functioning (PF), role limitations due to physical health (RP), bodily pain (BP), general health (GH), role limitations due to emotional problems (RE), vitality/energy and fatigue (VT), mental health (MH), social functioning (SF), and health change in the past year (HC). We categorised the first four components to yield the physical composite score and the latter four components to produce the mental composite score as Zhu et al. reported [27]. The scores varied between 0 and 100; the higher score, the better the quality of life is [28]. We used the Arabic model of the inquiry, which is valid and reliable [29]. Cronbach’s alpha for all subscales of the SF-36 for our study exceed alpha of 0.70. Face and content validity of the final version of instrument was discussed and evaluated by a panel of three researchers who are expert in research related to sleep and QOL for assessing the organization, appropriateness meaning of terms, clinical terminology, completeness, and logical sequence of the statements.

### Ethical approval

All rules of conduct were authorised by the Institutional Review Boards (IRB) before initiation of this study, and permission to interview the medical residents was granted by the local health authorities.

### Statistical analysis

All statistical analyses for our data were performed using the Statistical Package for Social Sciences version 15 (SPSS Inc., Chicago, IL, USA). Continuous data are presented as mean ± standard deviation (SD) or median (Q1–Q3; interquartile range) or frequency (percentage) for categorical variables. Data were tested for normality using the Kolmogorov–Smirnov test. In comparing two groups, we used Mann–Whitney U tests, and for comparing more than two groups, we used Kruskal–Wallis tests. We assessed the correlation between ESS and SF-36 domains using the Pearson’s correlation coefficient; *p* values < 0.05 were considered significant. Internal consistency reliability for all scales was assessed using Cronbach’s alpha.

## Results

### The characteristics of the resident doctors

Overall, 126 questionnaire were distributed, 112 (88.8%) were returned, 101 (80.1%) were accepted, and 11 (8.7%) were rejected for incompleteness (*n* = 8) or for respondents being general practitioners (*n* = 3). Of the 101 participants, 86 were male (85.1%) and 15 were female (14.9%), with a mean age of 28.5 ± 2.5. Table 1 shows the sociodemographic and general characteristics of the participants.

**Table 1:** Sociodemographic and general characteristics

Variables	n (%)
Age (per years)	
≤ 28	60(59.4)
> 28	41(40.6)
Sex	
Male	86(85.1)
Female	15(14.9)
Marital status	
Married	48(47.5)
Unmarried	53(52.5)
Governorate ^a^	
North	79(78.2)
Central	5(5.0)
South	17(16.8)
Living with family	
Yes	79(78.2)
No	22(21.8)
Body mass index ^b^	
Underweight/normal	39(38.6)
Overweight	50(49.5)
Obese	12(11.9)
Weight change during residency	
Increased	39(38.6)
Decreased	27(26.7)
No change	35(34.7)
Chronic diseases	
Hypertension	3(3.0)
Place of graduation ^c^	
Local	28(27.7)
Regional	52(51.5)
Western	21(20.8)
Working department	
General surgery	23(22.8)
Internal medicine	11(10.9)
Paediatrics	22(21.8)
Gynecology and obstetrics	14(13.9)
Anaesthesia	11(10.9)
Radiology	7(6.9)
Orthopaedics	13(12.9)
Residency years (per years)	
≤ 1	37(36.6)
> 1	64(63.4)
Years of experience (per years)	
≤ 2	33(32.7)
3–4	41(40.6)
> 4	27(26.7)

*n* frequency, *SD* standard deviation, *%* percentage

^a^ Governorates: North (Jenin, Nablus, Tulkarm, Qalqilya, Salfit, Tubas), Central (Jerusalem, Ramallah), South (Bethlehem, Hebron)

^b^ Body mass index: Underweight/normal (≤24), overweight (> 24 – < 30), obese (≥30)

^c^ Place of graduation: Local (Palestine), regional (Arab-world countries other than Palestine), western (remaining countries worldwide)

### Health-related quality of life in the participant residents

The means of the SF-36 subscales were calculated in Table 2, and the results, as shown in Additional file 1: Table S1 and Table S2, indicate a statistically significant difference in all of the subscale components with different variables at *p* < 0.05 except for mental health, which showed no significance. As for physical functioning, there is significance with the years of experience (*p* = 0.032). Role physical was significant in residents living with their family (*p* = 0.043). Role emotional shows significance with the residents’ governorates (*p* = 0.042), years of residency (*p* = 0.004), and years of experience (*p* = 0.010). Vitality has significance with gender (*p* = 0.019), age (*p* = 0.016), marital status (*p* = 0.021), and place of graduation (*p* = 0.004). Social functioning is significant with gender (*p* = 0.006) and place of graduation (*p* = 0.049). Body pain is associated with change in weight during residency (*p* = 0.039) and the country of graduation (*p* = 0.005). General health is affected by the country of graduation (*p* = 0.022) and years of experience (*p* = 0.044). Health change is affected by weight change during residency (*p* = 0.023). On the other hand, as Table 3 shows, the mental composite score (MCS) was significant with gender (*p* = 0.006) and the governorates of the residents (*p* = 0.020); the physical composite score (PCS) had no apparent significance.

**Table 2:** 36-Item Short Form Health Survey (SF-36)

Variables	Mean ± SD
Physical functioning	80.2 ± 22.3
Role limitations due to physical health	61.9 ± 38.2
Role limitations due to emotional problems	45.9 ± 42.9
Energy/fatigue	39.6 ± 16.7
Emotional well-being	50.6 ± 18.8
Social functioning	49.9 ± 24.7
Pain	68.7 ± 22.7
General health	61.0 ± 17.2
Health change	42.1 ± 19.7
PCS	67.9 ± 19.9
MCS	46.4 ± 19.6
Average total	55.5 ± 16.4

*n* frequency, *SD* standard deviation, *%* percentage, *PCS* physical composite score, *MCS* mental composite score

**Table 3:** PCS and MCS according to sociodemographic and general characteristics

	n (%)	PCS Median [Q1–Q3]	*p* value ^d^	MCS Median [Q1–Q3]	*p* value ^d^
Age (per years)					
≤ 28	60 (59.4)	70.4[50.2–87.4]	0.849 ^e^	41.0[28.3–61.9]	0.424 ^e^
> 28	41(40.6)	72.0[52.5–85.0]		44.3[33.0–65.5]	
Sex					
Male	86 (85.1)	71.6[51.7–87.1]	0.369 ^e^	45.3[32.2–64.6]	**0.006** ^**e**^
Female	15 (14.9)	62.0[49.5–80.0]		28.0[20.3–41.5]	
Marital status					
Married	48(47.5)	57.5[48.9–87.1]	0.570 ^e^	41.5[31.3–62.6]	0.957 ^e^
Unmarried	53(52.5)	72.0[52.3–86.0]		44.0[28.1–64.0]	
Governorate ^a^					
North	79(78.2)	72.0[52.5–85.8]		42.0[28.3–62.0]	**0.020** ^**f**^
Central	5(5.0)	48.3[36.6–82.9]		24.0[17.4–37.5]	
South	17(16.8)	70.0[46.9–89.8]	0.512 ^f^	59.0[35.3–70.1]	
Living with family					
Yes	79(78.2)	72.0[52.0–87.0]	0.103 ^e^	44.8[28.3–65.3]	0.248 ^e^
No	22(21.8)	58.5[45.9–77.5]		39.0[28.4–49.9]	
Body mass index ^b^					
Underweight/normal	39(38.6)	70.8[51.3–88.3]	0.368 ^f^	41.5[27.0–64.5]	0.899 ^f^
Overweight	50(49.5)	73.5[52.5–86.6]		42.6[32.1–63.1]	
Obese	12(11.9)	56.3[47.4–78.0]		43.6[31.8–61.9]	
Weight change during residency					
Increased	39(38.6)	72.5[49.5–88.3]	0.238 ^f^	49.5[32.0–67.3]	0.147 ^f^
Decreased	27(26.7)	59.5[48.3–80.8]		36.3[26.3–59.0]	
No change	35(34.7)	72.0[52.5–88.8]		44.8[32.0–65.3]	
Place of graduation ^c^					
Local	28(27.7)	70.0[51.6–86.7]	0.085 ^f^	41.4[26.4–57.6]	0.130 ^f^
Regional	52(51.5)	64.4[47.1–82.8]		40.4[28.3–64.1]	
Western	21(20.8)	82.5[62.6–90.4]		51.3[40.4–68.9]	
Working department					
General surgery	23(22.8)	62.5[50.0–86.3]	0.156 ^f^	38.3[26.0–53.8]	0.585 ^f^
Internal medicine	11(10.9)	87.0[60.8–90.0]		54.0[32.0–73.0]	
Paediatrics	22(21.8)	59.1[48.4–78.4]		43.4[30.9–58.4]	
Gynecology and obstetrics	14(13.9)	69.8[47.5–80.6]		35.9[27.8–65.6]	
Anaesthesia	11(10.9)	85.8[74.5–95.0]		49.5[40.8–64.8]	
Radiology	7(6.9)	72.0[52.5–83.3]		38.5[26.3–72.8]	
Orthopaedics	13(12.9)	70.8[52.3–89.4]		48.8[35.4–67.0]	
Residency years (per years)					
≤ 1	37(36.6)	72.0[50.4–88.1]		47.8[30.8–65.0]	0.196 ^e^
> 1	64(63.4)	68.8[51.3–85.0]	0.617 ^e^	41.0[28.3–58.1]	
Years of experience (per years)					
≤ 2	33(32.7)	72.0[51.0–88.1]		54.0[30.8–66.3]	0.149 ^f^
3–4	41(40.6)	60.8[49.1–82.5]	0.116 ^f^	40.0[28.3–53.5]	
> 4	27(26.7)	75.8[52.5–88.8]		41.0[27.8–71.0]	

*n* frequency, *%* percentage, *PCS* physical composite score, *MCS* mental composite score

^a^ Governorates: North (Jenin, Nablus, Tulkarm, Qalqilya, Salfit, Tubas), Central (Jerusalem, Ramallah), South (Bethlehem, Hebron)

^b^ Body mass index: Underweight/normal (≤24), overweight (> 24 – < 30), obese (≥30)

^c^ Place of graduation: Local (Palestine), regional (Arab-world countries other than Palestine), western (remaining countries worldwide)

^d^ The *p*-value is bold where it is less than the significance level cut-off of 0.05

^e^ Statistical significance of differences calculated using the Mann–Whitney U test

^f^ Statistical significance of differences calculated using the Kruskal–Wallis test

### The sleep characteristics of the resident doctors

The sleep features of the participants are detailed in Table 4. The ESS median is 9, and a score of ≥10 indicating daytime sleepiness is observed in 38 (37.6%) participants. The ESS is compared with sociodemographic and general characteristics to yield no significant association between them, as shown in Table 5. In comparing the ESS with the sleep characteristics, the only significant difference present is in having nightmares (*p* = 0.004), as Table 6 elaborates.

**Table 4:** Sleep characteristics

Variables	n (%)
Smoking	40(39.6)
Caffeine use	95(94.1)
Coffee (per ml)	
None	20(19.8)
Up to 100	8(7.9)
Up to 200	22(21.8)
Up to 300	31(30.7)
More than 300	20(19.8)
Tea (per ml)	
None	34(33.7)
Up to 100	33(32.7)
Up to 200	18(17.8)
Up to 300	11(10.9)
More than 300	5(5.0)
Energy drinks (per ml)	
None	87(86.1)
Up to 100	6(5.9)
Up to 200	3(3.0)
Up to 300	5(5.0)
Caffeine at night (times per week)	
None	34(33.7)
Once or twice	29(28.7)
Three to four	16(15.8)
More than four	22(21.8)
Non-shift sleep hours	
≤ 4 h	5(5.0)
4–6 h	61(60.4)
≥ 6 h	35(34.7)
Shift sleep hours	
≤ 4 h	85(84.1)
4–6 h	14(13.9)
≥ 6 h	2(2.0)
Sleeping pills use	4(4.0)
Time taken to fall asleep	
≤ 10mins	27(26.7)
10–30 min	35(34.7)
30–60 min	28(27.7)
> 60mins	11(10.9)
Waking up during sleep	73(72.3)
Wake up to eat	17(16.8)
Wake up due to noise	59(58.4)
Sleep walking	0(0.0)
Nightmares	51(50.5)
Epworth Sleepiness Scale	
< 10	63(62.4)
≥ 10	38(37.6)

**Table 5:** Epworth Sleepiness Scale according to sociodemographic and general characteristics

Variables	n (%)	ESS Median [Q1–Q3]	*P* value ^d^
Age (per years)			
≤ 28	60(59.4)	9.0[5.0–11.0]	0.670 ^e^
> 28	41(40.6)	8.0[6.5–11.0]	
Sex			
Male	86(85.1)	8.5[6.0–11.0]	0.958 ^e^
Female	15(14.9)	9.0[4.0–12.0]	
Marital status			
Married	48(47.5)	9.0[6.3–12.0]	0.067 ^e^
Unmarried	53(52.5)	8.0[5.0–11.0]	
Governorate ^a^			
North	79(78.2)	9.0[6.0–11.0]	0.861^f^
Central	5(5.0)	10.0[5.5–13.5]	
South	17(16.8)	8.0[5.5–13.5]	
Living with family			
Yes	79(78.2)	8.0[5.0–11.0]	0.063 ^e^
No	22(21.8)	10.0[7.0–12.3]	
Body mass index ^b^			
Underweight/normal	39(38.6)	8.0[5.0–11.0]	0.110 ^f^
Overweight	50(49.5)	9.0[7.0–12.0]	
Obese	12(11.9)	7.0[4.5–11.8]	
Weight change during residency			
Increased	39(38.6)	9.0[6.0–12.0]	0.300 ^f^
Decreased	27(26.7)	9.0[6.0–12.0]	
No change	35(34.7)	7.0[5.0–11.0]	
Place of graduation ^c^			
Local	28(27.7)	9.0[7.0–11.0]	0.650 ^f^
Regional	52(51.5)	8.5[6.0–11.0]	
Western	21(20.8)	7.0[4.0–11.5]	
Working department			
General surgery	23(22.8)	9.0[7.0–13.0]	0.755 ^f^
Internal medicine	11(10.9)	8.0[4.0–9.0]	
Paediatrics	22(21.8)	8.5[6.3–11.3]	
Gynecology and obstetrics	14(13.9)	9.0[7.0–10.3]	
Anaesthesia	11(10.9)	5.0[4.0–13.0]	
Radiology	7(6.9)	8.0[5.0–11.0]	
Orthopaedics	13(12.9)	9.0[4.0–11.5]	
Residency years (per years)			
≤ 1	37(36.6)	8.0[4.0–11.0]	0.064 ^e^
> 1	64(63.4)	9.0[7.0–11.0]	
Years of experience (per years)			
≤ 2	33(32.7)	8.0[4.0–10.0]	0.243 ^f^
3–4	41(40.6)	9.0[6.5–12.0]	
> 4	27(26.7)	9.0[6.0–12.0]	

*n* frequency, *%* percentage, *ESS* Epworth Sleepiness Scale

^a^Governorates: North (Jenin, Nablus, Tulkarm, Qalqilya, Salfit, Tubas), Central (Jerusalem, Ramallah), South (Bethlehem, Hebron)

^b^ Body mass index: Underweight/normal (≤24), overweight (> 24 – < 30), obese (≥30)

^c^ Place of graduation: Local (Palestine), regional (Arab-world countries other than Palestine), western (remaining countries worldwide)

^d^ The *p*-value is bold where it is less than the significance level cut-off of 0.05

^e^ Statistical significance of differences calculated using the Mann–Whitney U test

^f^ Statistical significance of differences calculated using the Kruskal–Wallis test

**Table 6:** Epworth Sleepiness Scale according to sleep characteristics

Variables	n (%)	ESS Median [Q1–Q3]	*P* value ^a^
Smoking			
Yes	40(39.6)	9.0[5.3–11.8]	0.728 ^b^
No	61(60.4)	8.0[6.0–11.0]	
Coffee (per ml)			
None	20(19.8)	7.5[6.3–9.8]	0.222 ^c^
Up to 100	8(7.9)	9.0[8.0–13.0]	
Up to 200	22(21.8)	8.5[6.5–12.0]	
Up to 300	31(31.7)	9.0[7.0–12.0]	
More than 300	20(19.8)	5.5[3.3–10.8]	
Tea (per ml)			
None	34(33.7)	8.0[4.0–11.3]	0.145 ^c^
Up to 100	33(32.7)	9.0[7.0–12.0]	
Up to 200	18(17.8)	8.0[4.3–11.3]	
Up to 300	11(10.9)	8.0[7.0–13.0]	
More than 300	5(5.0)	4.0[3.0–7.5]	
Energy drinks (per ml)			
None	87(86.1)	9.0[6.0–11.0]	0.661 ^c^
Up to 100	6(5.9)	8.0[3.5–14.3]	
Up to 200	3(3.0)	8.0[7.0–8.5]	
Up to 300	5(5.0)	7.0[2.0–10.0]	
Caffeine at night (times per week)			
No	34(33.7)	8.0[7.0–11.3]	0.371 ^c^
Once or twice	29(28.7)	8.0[5.0–12.0]	
Three to four	16(15.8)	9.0[7.5–11.0]	
More than four	22(21.8)	7.0[4.0–10.3]	
Non-shift sleep hours			
≤ 4 h	5(5.0)	11.0[7.5–14.0]	0.297 ^c^
4–6 h	61(60.4)	9.0[6.0–11.0]	
≥ 6 h	35(34.7)	8.0[4.0–10.0]	
Shift sleep hours			
≤ 4 h	85(84.1)	8.0[6.0–11.0]	0.568 ^c^
4-6 h	14(13.9)	9.0[4.8–12.5]	
≥ 6 h	2(2.0)	6.5[5.0–8.0]	
Sleeping pill use			
Yes	4(4.0)	13.5[4.0–15.5]	0.196 ^b^
No	97(96.0)	8.0[6.0–11.0]	
Time taken to fall asleep			
≤ 10mins	27(26.7)	9.0[7.0–11.0]	0.275 ^c^
10–30 min	35(34.7)	8.0[6.0–11.0]	
30–60 min	28(27.7)	8.0[6.0–12.0]	
> 60mins	11(10.9)	6.0[2.0–9.0]	
Waking up during sleep			
Yes	73(72.3)	9.0[6.0–11.0]	0.741 ^b^
No	28(27.7)	8.5[4.8–12.8]	
Wake up to eat			
Yes	17(16.8)	9.0[7.0–12.0]	0.492 ^b^
No	84(83.2)	8.5[5.3–11.0]	
Wake up due to noise			
Yes	59(58.4)	9.0[5.0–11.0]	0.953 ^b^
No	42(41.6)	8.5[7.0–12.0]	
Nightmares			
Yes	51(50.5)	9.0[8.0–12.0]	**0.004** ^**b**^
No	50(49.5)	7.0[4.0–10.3]	

*n* frequency, *SD* standard deviation, *%* percentage, *ml* millilitres, *h* hours, *mins* minutes

^a^ The *p*-value is bold where it is less than the significance level cut-off of 0.05

^b^ Statistical significance of differences calculated using the Mann–Whitney U test

^c^ Statistical significance of differences calculated using the Kruskal–Wallis test

### Quality of health and sleepiness

There is a significant correlation between sleepiness and quality of health among the residents as provided by the negative correlation between the ESS and the SF-36 scores of the physical functioning (*r* = − 0.397, *p* < 0.001), role limitations due to physical health (*r* = − 0.200, *p* = 0.045), body pain (*r* = − 0.209, *p* = 0.036), general health (*r* = − 0.392, *p* < 0.001), and health change (*r* = − 0.199, *p* = 0.046) subscales, as shown in Additional file 1: Table S2. There are also significant positive correlations between the subscales of the SF-36 themselves as proved in Additional file 1: Table S2. This is also manifested in correlating the ESS and PCS, as there is a significant negative correlation between them (*r* = − 0.351, *p* < 0.001), unlike the ESS and MCS, where no significant correlation is present (*r* = − 0.097, *p* = 0.334).

## Discussion

This study aimed to assess the residents’ sleepiness and their quality of life. Few studies have linked those two categories, and no such study has been conducted in Palestine. Hereby, valid Arabic models of both the SF-36 and the ESS were used to fulfill the purpose of this study [25, 30]. The data gave the impression that medical residents may have reduced quality of both physical and mental health, but the results of this study were unanticipated regarding physical impairment. Our population may have had associations in some of the physical subscales with certain sociodemographic and general characteristics, but the PCS was unable to demonstrate so. Contrary to the insignificant result of the PCS in our study, a poor lipid profile and harmful lifestyle were exhibited among residents in Pikovsky et al. study, which may be reflected in their physical health [10]. This inconsistency may be due to the large number of residents in the same department and an unequal work load among them at the above-mentioned hospitals.

Regarding the participants’ mental health, the MCS manifested a poorer mental status in residents coming from the central region (Ramallah and Jerusalem) and in female participants. Distance plays a role in this case, as residents from the central region usually do not take dormitories, unlike the southern population, according to Belayachi et al. [31].

Female doctors are more prone to mental health liability compared to male doctors, as indicated by Sundquist and Johansson [32].

Concerning the sleepiness of the participants, 38 (37.6%) were sleepy by scoring 10 or more on the ESS, but, contrary to our expectation, having nightmares is surprisingly the only single factor between both the sociodemographic and sleep features to yield a significant relationship with the ESS. To support our result, Paul et al. elaborated that nightmares are associated with significantly decreased quality of sleep [33]. A systematic review by Bolster and Rourke found that in seven studies concerning shift hours, restriction had no influence on the residents’ well-being, which could explain why there was no association between working hours and the ESS [34].

In the current study, correlating sleepiness with quality of life showed that daytime sleepiness leads to poor physical composite but, surprisingly, has an insignificant effect on the mental aspect according to the MCS. This finding is contrary to previous studies that suggested a worsening individual mood, depressive symptoms, and emotional exhaustion [17, 31].

Although this study is the first study of its kind to investigate the quality of life among resident doctors and their quality of sleep, several limitations that should be noted. First, the current study was limited by involving just the north of West Bank governmental hospitals i.e. Nablus, Jenin and Tulkarm, which led to a small sampling size, preventing generalisation. Second, being a cross-sectional type of study also made it difficult to know whether our outcome was affected prior to the residency period. Additionally, as a cross-sectional design was used, a causal relationship was difficult to infer. Third, the small number of female practitioners in the residency programmes is another drawback. Fourth, possibility that the scales were inaccurately filled in as they are self-reporting scales. Fifth, the exclusion of ophthalmology, otolaryngology, and emergency wards due to scarcity of residents also raised the susceptibility of bias. Sixth, ESS has low efficiency when used in evaluating sleepiness in general populations such as in our study [35, 36]. Finally, we did not use standardized and validated questionnaires to evaluate sleep quality and quantity; moreover sleep deprivation is evaluated only on number of hours of sleep and not by sleep logs or actigraphy. In this way none information is collected about possible co-occurrence of sleep disorders (e.g. snoring, obstructive sleep apnea, insomnia, restless legs syndrome etc) which may influence daytime sleepiness.

## Conclusions

This paper has examined independently the qualities of life and sleep of West Bank medical residents working in the northern hospitals as well as the relationship between them. It appeared that female residents and those from the central region had poorer quality of life in the mental aspect. The paper also indicated increased sleepiness in participants who experience nightmares. Moreover, this study identified that daytime sleepiness impacts the residents physically, leading to a decreased quality of life. Daytime sleepiness and improvement of quality of life require health promotion actions among medical residents. Therefore, there is a definite need to enhance current policies regarding the residency programme schedule and, if needed, to further restrict working hours, and increasing the number of residents during each shift. One of the practical implications that can be made to ameliorate the residents’ well-being is to promote better sleep hygiene by providing time and comfortable rooms for a nap, and stress-relief activities.

## Additional file

Additional file 1: **Table S1** and **Table S2.** SF-36 subscales scores according to sociodemographic and general characteristics, and Pearson correlation between Epworth Sleepiness Scale and SF-36 subscales. (DOC 114 kb)

## Abbreviations

ArESS: Arabic Version of the Epworth Sleepiness Scale
BMI: Body mass index
BP: Bodily pain
ESS: Epworth sleepiness scale
GH: General health
HC: Health change in the past year
IRB: Institutional review board
MCS: Mental composite score
MH: Mental health
PCS: Physical composite score
PF: Physical functioning
Q1–Q3: Interquartile range
RE: Role limitations due to emotional problems
REM: Rapid eye movement
RP: Role limitations due to physical health
SD: Standard deviation
SF: Social functioning
SF-36: 36-Item Short Form Health Survey
SPSS: Statistical Package for Social Sciences
VT: Vitality/energy and fatigue
