# Supplementary material for: Epworth sleepiness scale in medical residents: quality of sleep and its relationship to quality of life
Source: J Occup Med Toxicol. 2018 Jul 13;13:21. doi: 10.1186/s12995-018-0203-z (PMC6043987; doi:10.1186/s12995-018-0203-z)
Supplement: Supplementary file 1 — Table S1 and Table S2. SF-36 subscales scores according to sociodemographic and general characteristics, and Pearson correlation between Epworth Sleepiness Scale and SF-36 subscales. (DOC 114 kb) [file 12995_2018_203_MOESM1_ESM.doc]

**Additional file 1: Table S1 and Table S2:** SF-36 subscales scores according to sociodemographic and general characteristics, and Pearson correlation between Epworth Sleepiness Scale and SF-36 subscales

**Table S1** SF-36 subscales scores according to sociodemographic and general characteristics

| HC  Median[Q1-Q3] | GH  Median[Q1-Q3] | BP  Median[Q1-Q3] | SF  Median[Q1-Q3] | MH  Median[Q1-Q3] | VT  Median[Q1-Q3] | RE  Median[Q1-Q3] | RP  Median[Q1-Q3] | PF  Median[Q1-Q3] | n (%) |  |
| --- | --- | --- | --- | --- | --- | --- | --- | --- | --- | --- |
| 50.0[25.0-50.0]  50.0[25.0-50.0] | 60.0[46.3-73.8]  60.0[45.0-75.0] | 68.0[45.0-89.5]  68.0[55.0-95.0] | 44.0[25.0-63.0]  50.0[31.5-75.0] | 48.0[36.0-67.0]  52.0[40.0-62.0] | 35.0[25.0-48.8]  45.0[32.5-50.0] | 33.0[0.0-100.0]  33.0[0.0-100.0] | 75.0[25.0-100.0]  50.0[37.5-100.0] | 90.0[75.0-98.8]  85.0[70.0-97.5] | 60(59.4)  41(40.6) | **Age (per years)**  ≤28  >28 |
| *0.789* | *0.470* | *0.302* | *0.318* | *0.454* | ***0.016*** | *0.810* | *0.971* | *0.545* | ***P (sig.)*** ^e^ |  |
| 50.0[25.0-50.0]  25.0[25.0-50.0] | 60.0[45.0-75.0]  60.0[45.0-65.0] | 68.0[55.0-90.0]  55.0[45.0-80.0] | 50.0[38.0-75.0]  25.0[25.0-38.0] | 48.0[36.0-68.0]  44.0[28.0-52.0] | 40.0[30.0-50.0]  25.0[15.0-45.0] | 33.0[0.0-100.0]  0.0[0.0-67.0] | 75.0[25.0-100.0]  50.0[25.0-100.0] | 85.0[75.0-96.2]  85.0[70.0-100.0] | 86(85.1)  15(14.9) | **Sex**  Male  Female |
| *0.067* | *0.632* | *0.084* | ***0.006*** | *0.126* | ***0.019*** | *0.155* | *0.364* | *0.847* | ***P (sig.)*** ^e^ |  |
| 25.0[25.0-50.0]  50.0[25.0-50.0] | 62.5[45.0-75.0]  60.0[52.5-72.5] | 68.0[47.5-97.0]  68.0[51.5-90.0] | 50.0[28.8-63.0]  50.0[25.0-75.0] | 52.0[40.0-60.0]  44.0[32.0-70.0] | 40.0[35.0-50.0]  30.0[22.5-50.0] | 33.0[0.0-91.8]  67.0[0.0-100.0] | 62.5[25.0-100.0]  75.0[25.0-100.0] | 82.5[58.8-95.0]  90.0[80.0-100.0] | 48(47.5)  53(52.5) | **Marital status**  Married  Unmarried |
| *0.75* | *0.717* | *0.869* | *0.937* | *0.221* | ***0.021*** | *0.170* | *0.935* | *0.111* | ***P (sig.)*** ^e^ |  |
| 50.0[25.0-50.0]  25.0[25.0-50.0]  50.0[25.0-50.0] | 60.0[50.0-75.0]  45.0[27.5-77.5]  65.0[42.5-77.5] | 68.0[55.0-90.0]  55.0[18.0-79.0]  70.0[45.0-90.0] | 50.0[25.0-63.0]  25.0[6.5-44.0]  50.0[31.5-75.0] | 48.0[40.0-64.0]  36.0[24.0-62.0]  56.0[36.0-74.0] | 40.0[25.0-50.0]  25.0[15.0-40.0]  45.0[25.0-55.0] | 33.0[0.0-100.0]  0.0[0.0-33.0]  67.0[50.0-100.0] | 75.0[25.0-100.0]  25.0[12.5-100.0]  75.0[25.0-100.0] | 85.0[75.0-100.0]  80.0[60.0-97.5]  85.0[62.5-95.0] | 79(78.2)  5(5.0)  17(16.8) | **Governorates they come from** ^a^  North  Central  South |
| *0.575* | *0.751* | *0.377* | *0.078* | *0.459* | *0.204* | ***0.042*** | *0.807* | *0.577* | ***P (sig.)*** ^f^ |  |
| 50.0[25.0-50.0]  50.0[25.0-50.0] | 60.0[45.0-75.0]  55.0[45.0-67.5] | 68.0[55.0-90.0]  61.5[45.0-88.5] | 50.0[25.0-63.0]  44.0[25.0-66.0] | 48.0[36.0-68.0]  48.0[39.0-53.0] | 40.0[25.0-50.0]  40.0[28.8-45.0] | 33.0[0.0-100.0]  16.5[0.0-75.3] | 75.0[25.0-100.0]  50.0[18.8-81.3] | 90.0[75.0-100.0]  80.0[66.3-91.3] | 79(78.2)  22(21.8) | **Living with the family**  Yes  No |
| *0.131* | *0.190* | *0.438* | *0.650* | *0.586* | *0.566* | *0.138* | ***0.043*** | *0.107* | ***P (sig.)*** ^e^ |  |
| 50.0[25.0-50.0]  50.0[25.0-50.0]  25.0[25.0-50.0] | 60.0[50.0-80.0]  60.0[50.0-75.0]  50.0[45.0-73.8] | 68.0[45.0-78.0]  68.0[55.0-90.0]  66.5[55.0-100.0] | 50.0[25.0-63.0]  50.0[25.0-75.0]  50.0[38.0-63.0] | 44.0[32.0-64.0]  48.0[40.0-61.0]  60.0[37.0-71.0] | 40.0[25.0-50.0]  40.0[25.0-46.3]  45.0[31.3-57.5] | 33.0[0.0-100.0]  33.0[0.0-100.0]  0.0[0.0-33.0] | 75.0[25.0-100.0]  75.0[25.0-100.0]  50.0[25.0-68.8] | 90.0[70.0-100.0]  90.0[75.0-95.0]  75.0[55.0-85.0] | 39(38.6)  50(49.5)  12(11.9) | **Body mass index** ^b^  Underweight/Normal  Overweight  Obese |
| *0.514* | *0.534* | *0.635* | *0.849* | *0.519* | *0.615* | *0.909* | *0.350* | *0.094* | ***P (sig.)*** ^f^ |  |
| 25.0[25.0-50.0]  25.0[25.0-50.0]  50.0[50.0-50.0] | 55.0[45.0-75.0]  60.0[50.0-70.0]  65.0[55.0-80.0] | 78.0[55.0-100.0]  55.0[45.0-78.0]  68.0[55.0-90.0] | 50.0[38.0-63.0]  38.0[25.0-63.0]  50.0[38.0-63.0] | 52.0[40.0-64.0]  44.0[32.0-52.0]  52.0[32.0-72.0] | 45.0[35.0-55.0]  35.0[25.0-45.0]  40.0[25.0-50.0] | 33.0[0.0-100.0]  33.0[0.0-67.0]  33.0[0.0-100.0] | 75.0[25.0-100.0]  50.0[25.0-100.0]  75.0[50.0-100.0] | 85.0[70.0-95.0]  90.0[70.0-95.0]  85.0[75.0-100.0] | 39(38.6)  27(26.7)  35(34.7) | **Weight change during residency**  Increased  Decreased  No change |
| ***0.023*** | *0.093* | ***0.039*** | *0.395* | *0.074* | *0.130* | *0.496* | *0.233* | *0.723* | ***P (sig.)*** ^f^ |  |
| 50.0[25.0-50.0]  50.0[25.0-50.0]  50.0[25.0-50.0] | 60.0[46.3-73.8]  60.0[45.0-70.0]  75.0[57.5-80.0] | 61.5[45.0-89.5]  61.5[45.0-78.0]  88.0[73.0-100.0] | 38.0[25.0-63.0]  50.0[25.0-63.0]  63.0[38.0-75.0] | 44.0[33.0-52.0]  48.0[36.0-76.0]  52.0[44.0-68.0] | 30.0[25.0-45.0]  40.0[25.0-45.0]  50.0[40.0-60.0] | 33.0[0.0-100.0]  33.0[0.0-100.0]  33.0[0.0-100.0] | 75.0[25.0-100.0]  62.5[25.0-100.0]  75.0[50.0-100.0] | 85.0[76.3-100.0]  85.0[70.0-95.0]  95.0[75.0-100.0] | 28(27.7)  52(51.5)  21(20.8) | **Place of graduation** ^c^  Local  Regional  Western |
| *0.740* | ***0.022*** | ***0.005*** | ***0.049*** | *0.088* | ***0.004*** | *0.984* | *0.522* | *0.395* | ***P (sig.)*** ^f^ |  |
| 50.0[25.0-50.0]  50.0[25.0-50.0]  37.5[25.0-50.0]  25.0[25.0-50.0]  50.0[50.0-50.0]  50.0[50.0-50.0]  50.0[25.0-50.0] | 60.0[45.0-75.0]  70.0[55.0-80.0]  60.0[45.0-70.0]  57.5[48.8-66.3]  70.0[55.0-80.0]  70.0[55.0-75.0]  60.0[45.0-77.5] | 58.0[45.0-90.0]  87.0[68.0-100.0]  58.0[45.0-80.5]  56.5[55.0-88.5]  78.0[68.0-100.0]  58.0[45.0-78.0]  78.0[55.0-100.0] | 38.0[25.0-63.0]  38.0[25.0-63.0]  50.0[25.0-63.0]  38.0[25.0-53.3]  63.0[50.0-100.0]  75.0[38.0-88.0]  50.0[25.0-69.0] | 44.0[36.0-60.0]  56.0[48.0-76.0]  42.0[31.0-61.0]  52.0[37.0-70.0]  52.0[48.0-68.0]  44.0[40.0-84.0]  44.0[34.0-70.0] | 35.0[25.0-50.0]  45.0[25.0-55.0]  40.0[25.0-50.0]  37.5[25.0-50.0]  45.0[40.0-50.0]  35.0[25.0-40.0]  35.0[30.0-60.0] | 33.0[0.0-67.0]  67.0[0.0-100.0]  33.0[0.0-100.0]  33.0[0.0-100.0]  33.0[0.0-100.0]  0.0[0.0-100.0]  33.0[0.0-67.0] | 75.0[25.0-100.0]  100.0[25.0-100.0]  50.0[25.0-100.0]  50.0[25.0-100.0]  100.0[75.0-100.0]  50.0[0.0-100.0]  75.0[37.5-100.0] | 90.0[75.0-95.0]  95.0[80.0-100.0]  80.0[66.3-90.0]  82.5[70.0-92.5]  95.0[70.0-100.0]  100.0[85.0-100.0]  85.0[55.0-97.5] | 23(22.8)  11(10.9)  22(21.8)  14(13.9)  11(10.9)  7(6.9)  13(12.9) | **Working department**  General surgery  Internal medicine  Pediatrics  Gynecology and obstetrics  Anesthesia  Radiology  Orthopedics |
| *0.202* | *0.272* | *0.207* | *0.213* | *0.379* | *0.829* | *0.777* | *0.333* | *0.096* | ***P (sig.)*** ^f^ |  |
| 50.0[25.0-50.0]  50.0[25.0-50.0] | 60.0[50.0-77.5]  60.0[45.0-75.0] | 70.0[55.0-90.0]  68.0[45.0-90.0] | 50.0[31.5-75.0]  50.0[25.0-63.0] | 48.0[32.0-72.0]  48.0[40.0-60.0] | 35.0[25.0-52.5]  40.0[30.0-50.0] | 67.0[33.0-100.0]  33.0[0.0-67.0] | 75.0[25.0-100.0]  62.5[25.0-100.0] | 90.0[77.5-97.5]  85.0[70.0-98.75] | 37(36.6)  64(63.4) | **Residency years** (per years)  ≤1  >1 |
| *0.562* | *0.434* | *0.960* | *0.983* | *0.824* | *0.361* | ***0.004*** | *0.802* | *0.254* | ***P (sig.)*** ^e^ |  |
| 50.0[25.0-50.0]  50.0[25.0-50.0]  50.0[25.0-50.0] | 65.0[52.5-77.5]  55.0[45.0-70.0]  80.0[65.0-86.0] | 70.0[55.0-90.0]  65.0[45.0-89.0]  70.0[58.0-100.0] | 50.0[38.0-75.0]  38.0[25.0-63.0]  50.0[25.0-75.0] | 48.0[32.0-72.0]  48.0[40.0-60.0]  44.0[36.0-64.0] | 35.0[25.0-55.0]  40.0[25.0-45.0]  40.0[30.0-50.0] | 67.0[33.0-100.0]  0.0[0.0-83.5]  33.0[0.0-67.0] | 75.0[25.0-100.0]  50.0[25.0-100.0]  100.0[25.0-100.0] | 90.0[80.0-100.0]  80.0[70.0-90.0]  90.0[80.0-100.0] | 33(32.7)  41(40.6)  27(26.7) | **Years of experience** (per years)  ≤2  3-4  >4 |
| *0.885* | ***0.044*** | *0.239* | *0.172* | *0.990* | *0.545* | ***0.010*** | *0.216* | ***0.032*** | ***P (sig.)*** ^f^ |  |

*n* frequency, *%* percentage, *PF* Physical functioning, *RE* Role limitations due to physical health, *BP* Bodily pain, *GH* General health, *RE* Role limitations due to emotional problems, *VT* Vitality/Energy and fatigue, *MH* Emotional well-being/ Mental health, *SF* Social functioning, *HC* Health change in the past year, *Q1* first quartile, *Q3* third quartile

^a^ **Governorates:** North (Jenin, Nablus, Tulkarm, Qalqilya, Salfit, Tubas), Central (Jerusalem, Ramallah), South (Bethlehem, Hebron)

^b^ **Body mass index**: Underweight/Normal (≤24), Overweight (>24 - <30), Obese (≥30**)**

^c^ **Place of graduation:** Local (Palestine), Regional (Arab world countries other than Palestine), Western (Remaining countries worldwide)

^d^ The p-value is bold where it is less than the significance level cut-off of 0.05.

^e^ Statistical significance of differences calculated using the Mann-Whitney U test

^f^ Statistical significance of differences calculated using the Kruskal-Wallis test

**Table S2** Pearson correlation between Epworth Sleepiness Scale and SF-36 subscales

|  |  | **Physical Functioning** | **Role limitations due to physical health** | **Role limitations due to emotional health** | **Energy/fatigue** | **Emotional well-being** | **Social functioning** | **Pain** | **General health** | **Health change** |
| --- | --- | --- | --- | --- | --- | --- | --- | --- | --- | --- |
| **Epworth Sleepiness Scale score** | Pearson Correlation | -.397 | -.200 | -.044 | -.036 | -.091 | -.139 | -.209 | -.392 | -.199 |
|  | Sig. (2-tailed) | **<0.001** | **0.045** | 0.662 | 0.723 | 0.366 | 0.165 | **0.036** | **<0.001** | **0.046** |
| **Physical Functioning** | Pearson Correlation |  | .448 | .224 | .011 | .161 | .202 | .318 | .504 | .283 |
|  | Sig. (2-tailed) |  | **<0.001** | **0.024** | 0.915 | 0.109 | **0.043** | **0.001** | **<0.001** | 0.004 |
| **Role limitations due to physical health** | Pearson Correlation |  |  | .524 | .254 | .311 | .389 | .581 | .542 | .235 |
|  | Sig. (2-tailed) |  |  | **<0.001** | **0.010** | **0.002** | **<0.001** | **<0.001** | **<0.001** | **0.018** |
| **Role limitations due to emotional health** | Pearson Correlation |  |  |  | .350 | .361 | .418 | .470 | .284 | .089 |
|  | Sig. (2-tailed) |  |  |  | **<0.001** | **<0.001** | **<0.001** | **<0.001** | **0.004** | 0.375 |
| **Energy/fatigue** | Pearson Correlation |  |  |  |  | .589 | .457 | .415 | .225 | .275 |
|  | Sig. (2-tailed) |  |  |  |  | **<0.001** | **<0.001** | **<0.001** | **0.024** | **0.005** |
| **Emotional well-being** | Pearson Correlation |  |  |  |  |  | .371 | .465 | .376 | .220 |
|  | Sig. (2-tailed) |  |  |  |  |  | **<0.001** | **<0.001** | **<0.001** | **0.027** |
| **Social functioning** | Pearson Correlation |  |  |  |  |  |  | .541 | .389 | .089 |
|  | Sig. (2-tailed) |  |  |  |  |  |  | **<0.001** | **<0.001** | 0.374 |
| **Pain** | Pearson Correlation |  |  |  |  |  |  |  | .517 | .173 |
|  | Sig. (2-tailed) |  |  |  |  |  |  |  | **<0.001** | 0.083 |
| **General health** | Pearson Correlation |  |  |  |  |  |  |  |  | .257 |
|  | Sig. (2-tailed) |  |  |  |  |  |  |  |  | **0.009** |
